# Epinastine Eyelid Cream as a Practical Option for Patients With Glaucoma on Multidrug Topical Therapy: A Case Report

**DOI:** 10.7759/cureus.87589

**Published:** 2025-07-09

**Authors:** Yui Nishijima, Daisuke Hasegawa, Tatsuya Mimura

**Affiliations:** 1 Department of Ophthalmology, Tsurumi University School of Dental Medicine, Kanagawa, JPN; 2 Department of Ophthalmology, Keio University School of Medicine, Tokyo, JPN; 3 Department of Ophthalmology, Nerima Station West Eye Clinic, Tokyo, JPN

**Keywords:** adherence, allergic conjunctivitis, epinastine, eye drops, eyelid cream, glaucoma

## Abstract

Patients with glaucoma often require long-term use of multiple ophthalmic medications, which can make adherence to additional treatments, such as anti-allergic eye drops for seasonal allergic conjunctivitis, particularly challenging. Moreover, corticosteroid eye drops or ointments, commonly used to manage ocular allergy, are generally avoided in these patients due to the risk of increasing intraocular pressure. We report a case in which a patient with glaucoma, already undergoing multiple topical therapies, experienced recurrent allergic conjunctivitis and blepharitis each spring. The use of conventional anti-allergic eye drops had limited efficacy due to poor adherence associated with the complexity of the treatment regimen. To address this, a newly introduced epinastine cream was applied to the eyelids once daily as an alternative treatment. Following this change, the patient's allergic ocular symptoms improved substantially, with enhanced treatment compliance and no adverse effects on intraocular pressure during follow-up. This case suggests that non-ocular-drop formulations, such as eyelid creams, may offer a practical and effective approach for managing allergic eye disease in patients with glaucoma who are already on complex topical regimens.

## Introduction

Glaucoma is a chronic, progressive optic neuropathy that requires long-term management, and the standard treatment typically involves the use of multiple topical medications to control intraocular pressure (IOP) [1]. In recent years, the number of cases requiring combination therapy with prostaglandin analogs, β-blockers, carbonic anhydrase inhibitors, α2-adrenergic agonists, and Rho-associated coiled-coil containing protein kinase (ROCK) inhibitors has been increasing. ROCK inhibitors are novel glaucoma drugs that promote aqueous humor outflow through the fibrovascular zone. This has led to concerns that the growing number of eye drops may negatively impact patient adherence [2].

In East Asia, including Japan, seasonal allergic conjunctivitis caused by cedar and cypress pollen is common, and patients with glaucoma are no exception. Symptoms of allergic conjunctivitis (itching, redness, discomfort, etc.) have a significant impact on the quality of daily life. The addition of anti-allergic eye drops for seasonal allergic conjunctivitis to an already complex multidrug glaucoma regimen further complicates treatment and often results in reduced adherence to anti-allergic therapy [2]. Furthermore, poor adherence to anti-allergic eye drops may be attributed to complex dosing schedules and side effects from preservatives used in these formulations [3].

Given these challenges, alternative treatment approaches using non-drop formulations are needed. Topical corticosteroid eye drops and ophthalmic ointments have been widely used for their potent anti-inflammatory effects in the treatment of conditions such as dermatitis and blepharitis. However, prolonged application of corticosteroids around the eye may allow the drug to penetrate the intraocular tissues via the cornea and conjunctiva, potentially leading to an elevation in IOP and the development of steroid-induced glaucoma. This risk is of particular concern in patients with preexisting glaucoma, as further increases in IOP can result in severe and irreversible visual impairment. Therefore, careful consideration must be given to the choice of therapeutic agents in such patients [4]. Recently, 0.05% epinastine eyelid cream was launched in Japan as a novel topical therapy. Epinastin is both a histamine H1 receptor antagonist and a mast cell stabilizer. This cream, applied to the eyelid skin, is absorbed locally and has been reported to improve eyelid dermatitis and allergic conjunctivitis symptoms. The efficacy and safety of this formulation have been demonstrated in a phase III clinical trial [5]. The trial showed that once-daily eyelid application significantly reduced eyelid inflammation, conjunctival hyperemia, and ocular itching, with efficacy comparable to or greater than conventional anti-allergic eye drops.

Herein, we report a case of a patient with glaucoma undergoing multidrug eye drop therapy, in whom switching from anti-allergic eye drops to epinastine eyelid cream led to marked improvement in adherence and rapid relief of blepharitis and conjunctivitis symptoms.

## Case presentation

The patient was a 40-year-old man with no notable systemic diseases or family history of glaucoma. He was not taking any systemic medications. The patient had no history of allergic diseases, including asthma or atopic dermatitis, and had not been taking any oral antiallergic medications. Since the age of 10, he had suffered from seasonal allergic conjunctivitis associated with pollen allergy as well as perennial allergic conjunctivitis, both of which had been well controlled with a single anti-allergic eye drop. He had no history of using steroid eye drops or ophthalmic ointments.

**Figure 1:**
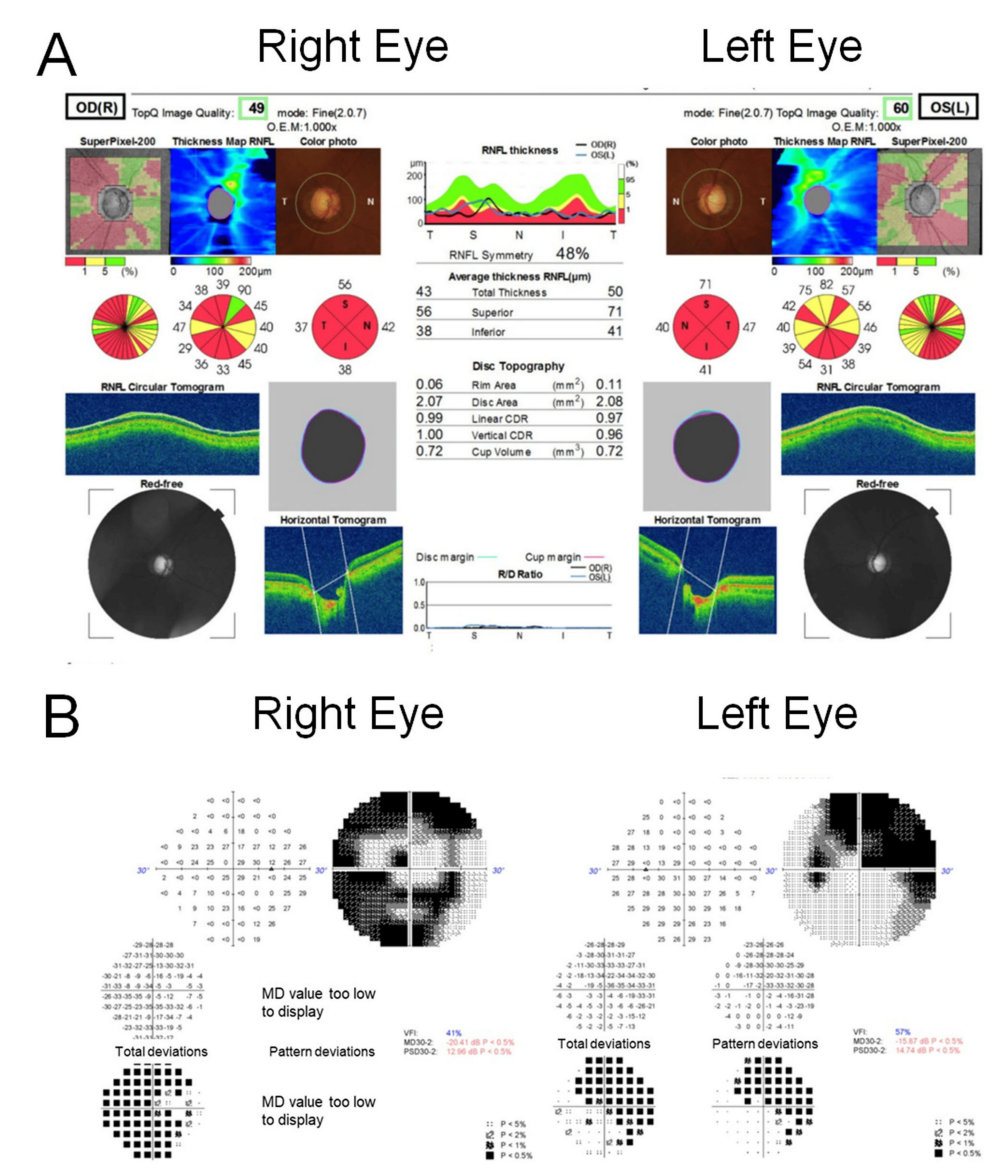
Latest ocular findings in a 40-year-old man undergoing glaucoma treatment. (A) Optical coherence tomography (OCT) images of both eyes showing thinning of the retinal nerve fiber layer. (B) Humphrey visual field test results of both eyes demonstrating marked visual field constriction consistent with glaucoma.

At age 30, glaucoma was suspected during a routine health screening, and he was referred to our hospital. His initial IOP was 21 mmHg in both eyes, and subsequent measurements showed IOP ranging from 21 to 27 mmHg bilaterally. Visual field testing revealed constriction, while head MRI findings were unremarkable. He was diagnosed with bilateral open-angle glaucoma. Treatment was initiated with latanoprost once daily. As visual field progression was observed, additional glaucoma eye drops were gradually introduced. From age 38 to the present, his regimen has included bimatoprost, dorzolamide/timolol fixed combination, brimonidine, and ripasudil. With this multidrug therapy, his IOP has been maintained at 15-18 mmHg in both eyes. The most recent optical coherence tomography (OCT) images (Figure 1A) and Humphrey visual field test results (Figure 1B) are shown. The patient's best-corrected visual acuity was 20/20 in the right eye with a refraction of -4.75/-0.25 × 165°, and 20/15 in the left eye with a refraction of -3.25/-0.75 × 170°. The corneas were clear, with no evidence of epithelial erosion.

Since the age of 30, he had experienced recurrent corneal epithelial erosion, allergic conjunctivitis, and blepharitis each spring due to pollen allergy. Epinastine hydrochloride LX 0.1% eye drops (twice daily) were prescribed for allergic conjunctivitis. However, adherence to the anti-allergic eye drops decreased due to the increased number of medications required for glaucoma, and a sufficient therapeutic effect was not achieved.

**Figure 2:**
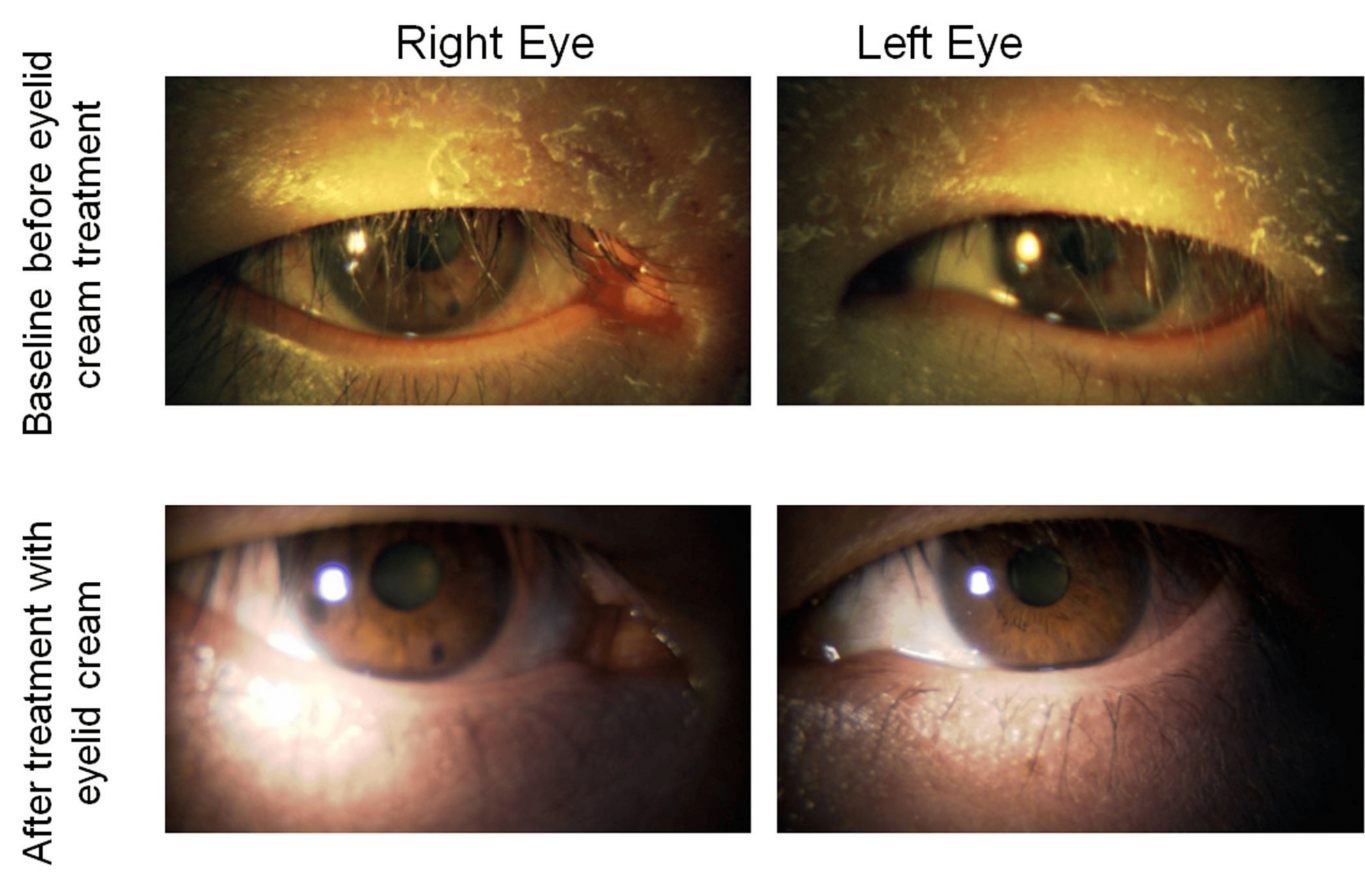
Eyelid findings before and after eyelid cream treatment. (A) Eyelid findings during treatment with epinastine eye drops, showing marked eyelid dermatitis and allergic conjunctivitis. (B) Eyelid findings two weeks after initiating epinastine eyelid cream, showing clear improvement of blepharitis and conjunctivitis symptoms.

In the spring at age 40, he developed marked eyelid dermatitis that did not improve with antiallergic eye drops (Figure 2A). Therefore, his treatment was switched from anti-allergic eye drops to 0.05% epinastine eyelid cream (applied once daily at night). As a result, adherence to anti-allergic treatment improved significantly, and nightly application was continued regularly. Two weeks after starting the eyelid cream, his blepharitis and allergic conjunctivitis symptoms showed clear improvement (Figure 2B). No recurrence has been observed over the following 12 months. His glaucoma eye drop regimen has also been maintained successfully, with IOP stabilized at 15-17 mmHg in both eyes.

## Discussion

This case describes a patient with glaucoma undergoing multidrug topical therapy in whom switching from anti-allergic eye drops to an epinastine eyelid cream improved treatment adherence and resulted in successful control of allergic conjunctivitis and blepharitis. The case highlights the importance of reducing treatment burden and improving adherence in the management of two chronic conditions: glaucoma and allergic conjunctivitis.

Glaucoma is a chronic disease that requires long-term treatment to preserve visual function, and the mainstay of therapy involves the use of multiple topical medications to lower intraocular pressure [6]. However, multidrug regimens increase patient burden, and it is well known that the number and frequency of eye drops can negatively affect adherence [7]. As seen in this case, adding anti-allergic eye drops to the glaucoma regimen further complicates treatment, potentially leading to reduced adherence to anti-allergic therapy itself.

In this patient, the use of four different glaucoma medications led to decreased frequency of anti-allergic eye drop use, making it difficult to control eyelid inflammation and corneal epithelial damage during the pollen season. Switching to once-daily epinastine 0.05% eyelid cream applied at night significantly improved adherence to anti-allergic therapy and provided rapid relief of symptoms. This approach proved useful in delivering effective anti-allergic treatment without adding to the burden of additional eye drop instillations.

Epinastine eyelid cream is a novel formulation recently introduced in Japan. It is absorbed through the eyelid skin and has been reported to improve symptoms of blepharitis and allergic conjunctivitis [5,8]. In this case, symptom relief was observed within two weeks of starting the cream, and no recurrence was noted during 12 months of follow-up, suggesting its potential contribution to long-term symptom control.

Furthermore, the course of this case suggests that introducing non-drop formulations may help reduce the overall treatment burden for patients and support adherence to glaucoma therapy. In patients on multidrug glaucoma regimens, increased treatment complexity can lead to discontinuation or suboptimal use of anti-allergic therapy, increasing the risk of corneal damage and subsequent visual impairment. This case illustrates that non-drop formulations such as eyelid cream could be a practical option to break this vicious cycle.

On the other hand, this is a single case report, and further investigation is needed to evaluate the long-term safety of epinastine eyelid cream and its efficacy across a broader patient population. In particular, many patients with glaucoma also have coexisting corneal epithelial disorders or dry eye disease, and further studies are warranted to clarify the benefits and potential side effects of eyelid cream in these contexts. Future randomized controlled trials and large-scale case series are needed to build evidence for new treatment approaches that could complement or replace conventional eye drop therapy.

## Conclusions

The use of epinastine eyelid cream may offer a new direction in managing allergic eye symptoms in patients with glaucoma. Its unique formulation could help simplify treatment regimens and support better long-term care strategies for complex ocular conditions.
